# Supplementary material for: Physical Mapping of QTL in Four Spring Wheat Populations under Conventional and Organic Management Systems. I. Earliness
Source: Plants (Basel). 2021 Apr 23;10(5):853. doi: 10.3390/plants10050853 (PMC8144964; doi:10.3390/plants10050853)
Supplement: Supplementary file 1 [file plants-10-00853-s001.zip › Supplementary files corrected 22Apr2021/Figure S1.pptx]

## Slide 1
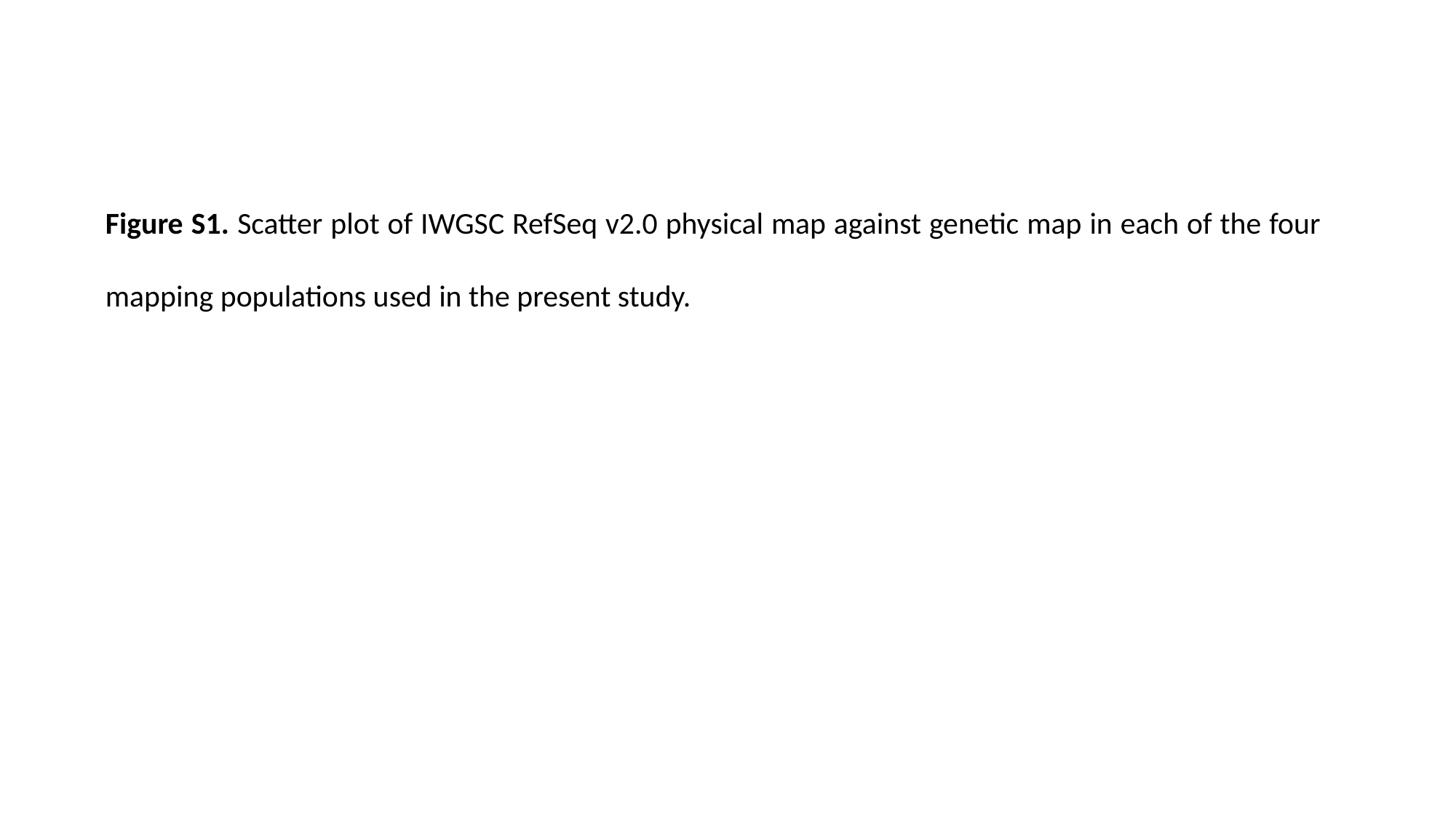

Figure S1. Scatter plot of IWGSC RefSeq v2.0 physical map against genetic map in each of the four mapping populations used in the present study.

## Slide 2
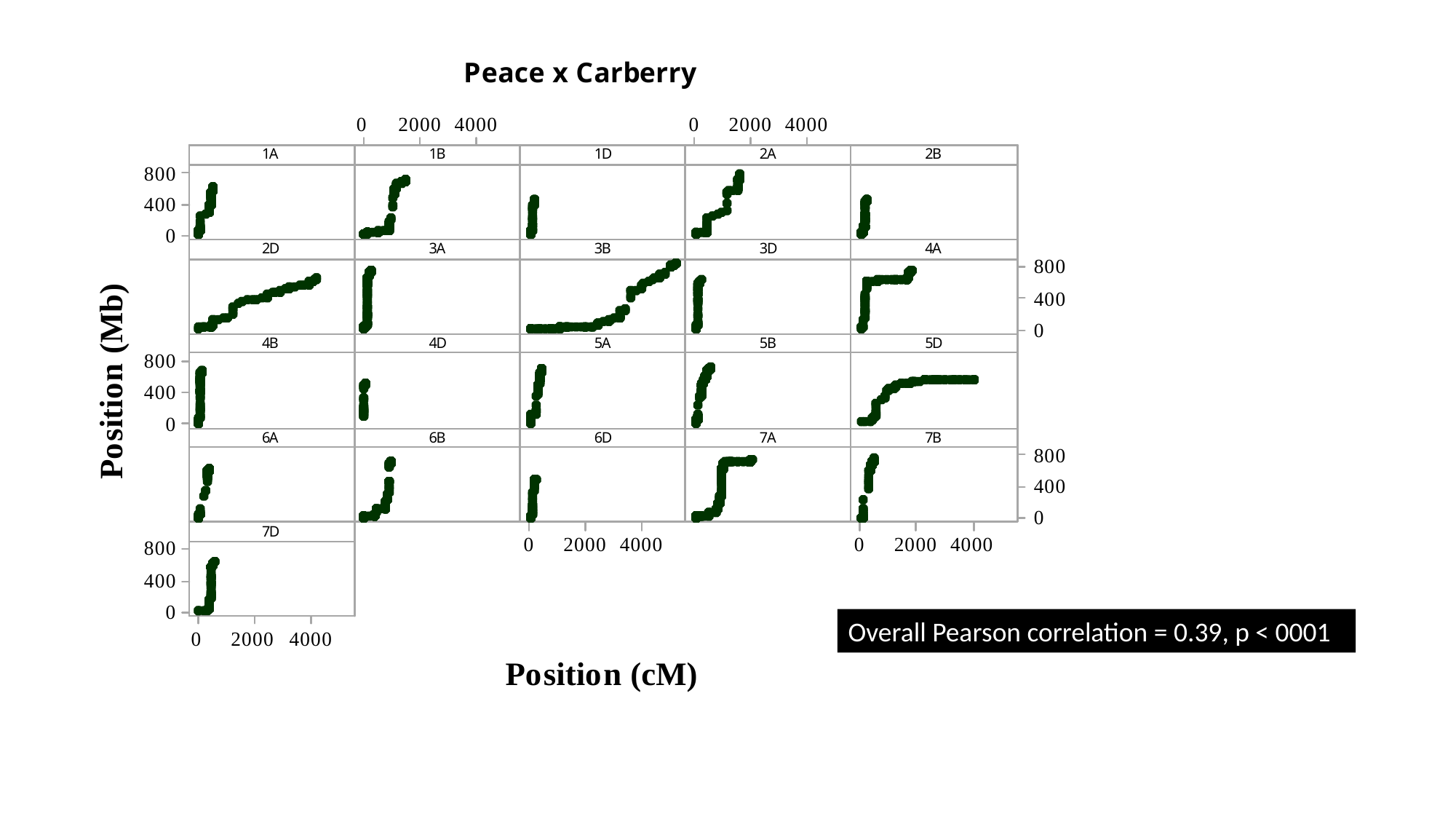

Overall Pearson correlation = 0.39, p < 0001

## Slide 3
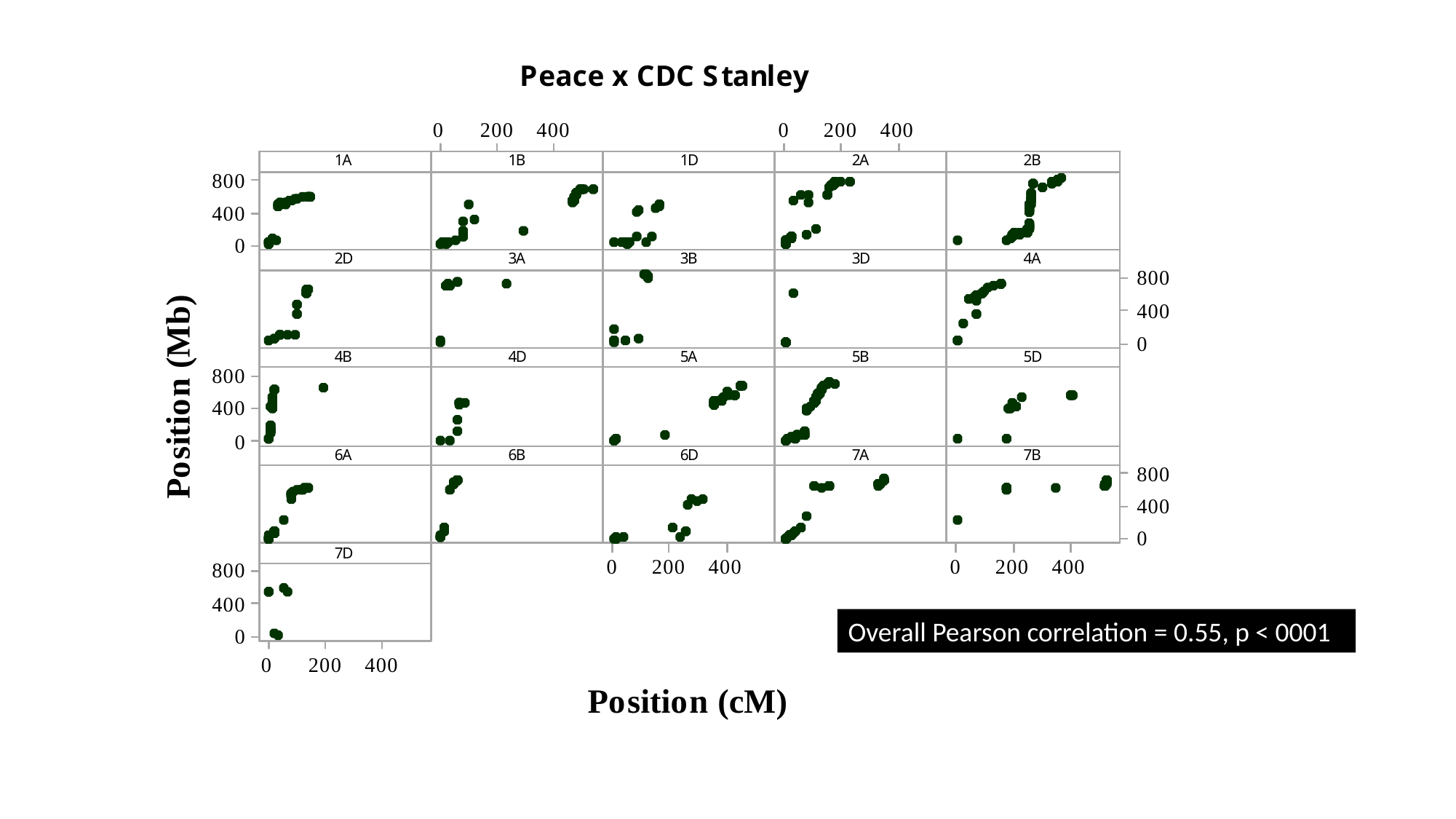

Overall Pearson correlation = 0.55, p < 0001

## Slide 4
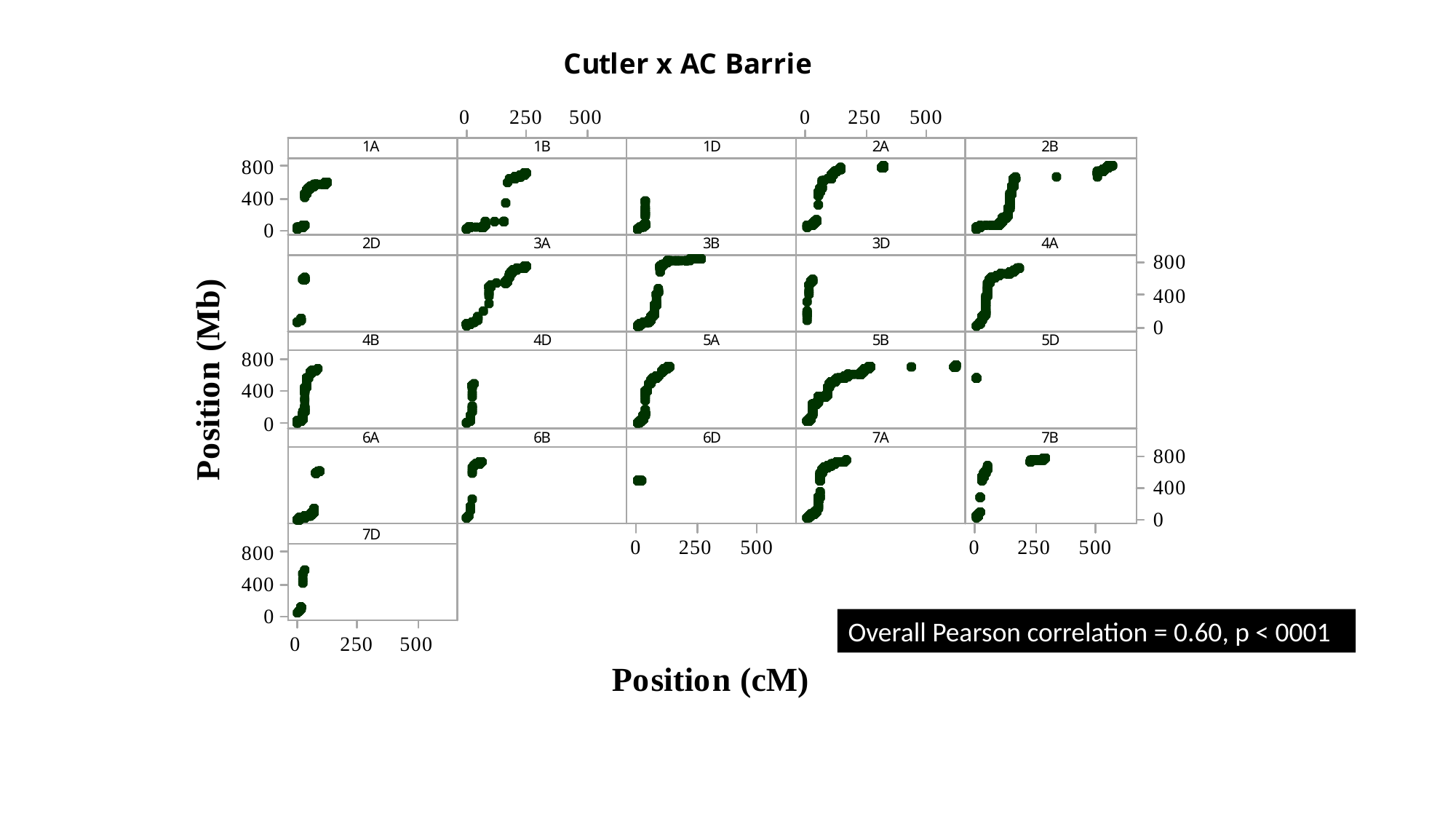

Overall Pearson correlation = 0.60, p < 0001

## Slide 5
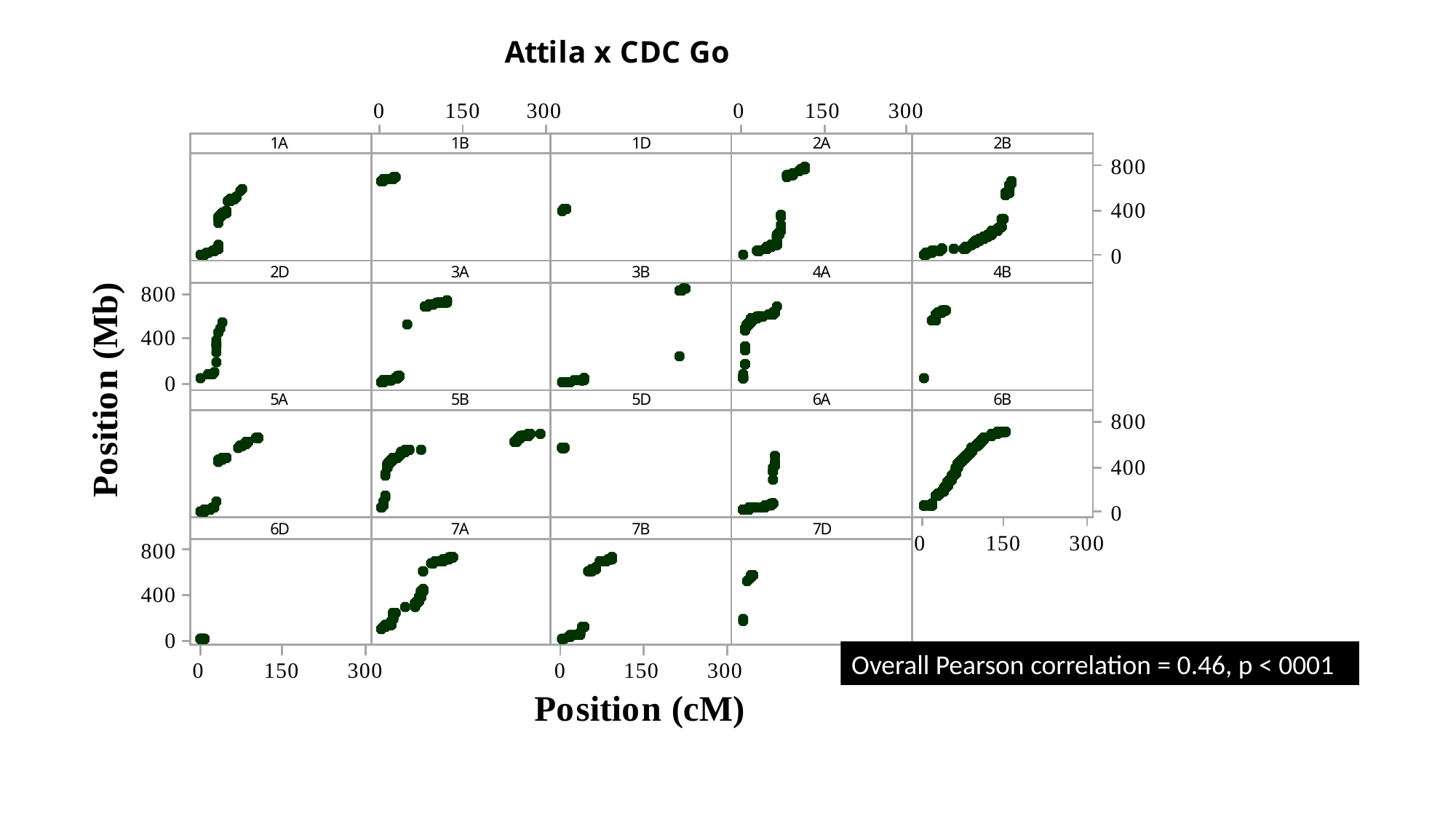

Overall Pearson correlation = 0.46, p < 0001
